# The Market Value of Hospital Service

**Published:** 1915-09-04

**Authors:** 


### THE MARKET VALUE OF HOSPITAL SERVICE.

It is with sincere sorrow, a sorrow that will be shared by every knowledgeable and competent worker of responsibility connected with most British hospitals worthy of the name, that we have to announce that Mr. W. G. Carnt has resigned the office of superintendent and secretary of the Manchester Royal Infirmary, owing to a complete breakdown in health, due initially to continuous overwork and overstrain in the cause of the great institution, to the interests of which he has devoted many of the best years of his life. On another page we deal fully with the past history and work of Mr. Carnt. His whole-hearted devotion and unselfish labours in the cause of the sick merit the grateful recognition in some practical form, under the leadership of the infirmary authorities, of the people of Manchester and the surrounding districts. Those of us who have watched and followed the initiative and work which Mr. Carnt has so well done, recognise him as a man of no common type, whose labours must live and bear fruit long after his inspiring leadership has been withdrawn from the centre in which he was the directing mind. Mr. Carnt will carry with him in his retirement the heartfelt sympathy of the whole hospital world. We all hope that he may, in God's good providence, recover his health, and be spared to enjoy many years of further usefulness when he has had sufficient time to rest his system and restore his energies and constitution. It is a grievous thing that so trusted and tried a helper in every department of hospital administration should be compelled to give up at a comparatively early age the work to which his whole soul has been devoted during many strenuous years.

The committee of the Royal Infirmary have reluctantly, and with the deepest regret, accepted the resignation of their general superintendent and secretary. They recognise that his services have proved invaluable to the institution, and they record the serious loss which it will sustain in being deprived of them, and the debt of gratitude which is due to him for his loyal, able, and energetic work on its behalf. We cannot suppose that the board of management will rest content with this emphatic declaration of the value of Mr. Carnt's services. For, as the *Manchester Courier* states, "Mr. Carnt could never be, and never has been, the mere executive officer of the board of management, and has certainly sacrificed his health to his devotion to the welfare of the Royal Infirmary. . . . Undoubtedly such yeoman work deserves more than the passing recognition of a resolution." What form the recognition of these services will take has yet to be determined. It must, of course, include the granting of an adequate pension, and we should hope also that Mr. Carnt's portrait will be painted by some eminent artist and hung in the board-room, with a suitable tablet commemorating his work.

During nearly a quarter of a century Mr. Carnt has devoted himself to the improvement and extended usefulness and efficiency of great voluntary hospitals. This period has produced many changes, and a revolution in treatment and in the means and methods both of medical and surgical practice and requirements. A great modern hospital has become a wonderful centre of organised efficiency, the upkeep, maintenance, and extension of which can only be sustained and provided under the direction of men specially trained to fit them to hold with success the higher offices. The work of the official head of a great London hospital is often arduous, but few positions in the hospital world require greater gifts or more special training and devotion than that of the general superintendent and secretary to the Manchester Royal Infirmary. It is matter for regret that at present there is no adequate system in existence in Great Britain for the selection and training of men of the highest character and abilities to fill the greater positions in the hospital field. This need is emphasised by the knowledge that overwork has been the bedrock cause of the loss to Manchester of Mr. Carnt's services.

We hope that the Board will seriously consider two important points which are not covered in the advertisements inviting applicants to fill the vacant post. One is that a great hospital like that at Manchester should protect itself by securing, through the appointment of a deputy superintendent, that its chief administrative officer shall not in future suffer from overstrain or be liable to sacrifice his health by devotion to his duties. The second point is that the importance and size of the Royal Infirmary, Manchester, call for a considerable increase in the salary offered. It may be argued that Mr. Carnt has brought the whole administration to the highest point of efficiency, so that the work of his successor will be simplified and made comparatively easy, that the present staff is excellent, and that practically all the special work which had to be done is now completed. Hence an ordinary man with some technical training, if appointed, could carry on the work. Experience, however, teaches that the best organised institutions speedily degenerate in such circumstances when the directing mind and initiative are withdrawn. After all, our great hospitals to succeed must be conducted on business principles, and it is not business to pay the higher officials less than the business value of their services. It is false economy and bad business for hospital governors to pay less than the market value for the highest service. To maintain its position in the hospital world Manchester should take infinite pains to secure the very best man available to succeed Mr. Carnt. A salary of £700 a year is not likely to provide such a successor. We do not doubt that a multitude of applications will be received, but Manchester needs the man, not the multitude.

